# A Multivariate RSM–PLS Framework and HPLC Polyphenolic Profiling for Characterizing Distinct Extraction Signatures in Pressurized Liquid vs. Conventional Stirring Extraction of Asteraceae Species

**DOI:** 10.3390/antiox15070789

**Published:** 2026-06-24

**Authors:** Aggeliki Alibade, Vassilis Athanasiadis, Martha Mantiniotou, Eleni Bozinou, Stavros I. Lalas

**Affiliations:** Department of Food Science and Nutrition, University of Thessaly, Terma N. Temponera Street, 43100 Karditsa, Greece; alimpante@uth.gr (A.A.); vaathanasiadis@uth.gr (V.A.); mmantiniotou@uth.gr (M.M.); empozinou@uth.gr (E.B.)

**Keywords:** *Solidago virgaurea*, *Tussilago farfara*, *Helichrysum stoechas*, PLE, response surface methodology, Box–Behnken design, bioactive compounds, antioxidant activity, HPLC-DAD, chemometrics

## Abstract

The current research investigates the extraction efficiency of an emerging green technology, pressurized liquid extraction (PLE), compared to traditional stirring extraction (STE) in order to recover higher antioxidant capacity from three plant species of the Asteraceae family, namely *Solidago virgaurea*, *Tussilago farfara*, and *Helichrysum stoechas*. The optimal PLE conditions were achieved through a combined response surface methodology (RSM) approach. The resulting optimized PLE parameters (40% ethanol, 160 °C, 25 min, 1700 psi) were experimentally verified and directly contrasted with STE (40% ethanol, 80 °C, 60 min, 500 rpm). Despite having the same solvent polarity, the two methods showed significant variations in mass transfer kinetics and heat intensity. Across all species, PLE significantly boosted the ascorbic acid antioxidant capacity (*p* < 0.05), thereby showing enhanced recovery of compounds that contribute to the overall antioxidant capacity. STE generated noticeably increased total polyphenolic content and DPPH radical scavenging activity (*p* < 0.05), indicating that some phenolic subclasses might be susceptible to PLE at higher temperatures. Values for ferric-reducing antioxidant power were largely similar among approaches. Overall, PLE was shown to be highly effective in maximizing the total antioxidant capacity in shorter extraction times, while STE can better preserve specific polyphenolic fractions, as demonstrated through analysis of the optimal extracts by HPLC-DAD. The integration of experimental validation with chemometric modeling supports the reliability and practical applicability of the optimized PLE protocol.

## 1. Introduction

Conventional extraction techniques have limitations, such as low yield, high solvent and energy consumption, and extended processing times, reducing viability and increasing costs [1]. The need to reduce environmental effects has received a lot of attention in recent years. To reduce negative effects on the environment, many industries are implementing innovative, environmentally friendly extraction techniques. In this context, PLE emerges as a superior alternative technique, outperforming conventional methods in terms of extractive capacity and operational efficiency [2], as it relies on high temperatures (50–200 °C) and pressures (35–200 bar), which enhances mass transfer and solubility of target compounds, leading to effective higher extraction yields [3,4]. At the same time, the increase in temperature leads to a decrease in the viscosity and surface tension of the solvent, as well as an increase in the diffusion coefficient, which promotes its penetration into the sample. Furthermore, the enhanced solubility of the target compounds under these conditions contributes to the acceleration of the extraction mobility [5]. PLE is posed as an easy-to-use method for the isolation of secondary metabolites and contaminants from natural matrices, offering advantages such as speed, reproducibility, and automation [5,6].

One of the largest and most important plant families is Asteraceae, whose many species have been used in traditional medicine since ancient times. Their use dates back thousands of years, as many of these plants have remarkable therapeutic indications. Traditionally, plants of the Asteraceae family have been used to relieve the common cold and cough [7], as well as to treat respiratory and digestive disorders [8,9,10,11]. The study of these plants has mainly focused on their chemical composition and information on their individual components, such as phenolic compounds, terpenoids, flavonoids, and essential oils [12]. Studies have revealed a variety of biological activities, such as antimicrobial, anti-inflammatory, hepatoprotective, antidiabetic, antioxidant, antibacterial, anticancer, and antiparasitic activities [10,13,14,15,16]. Knowledge about the therapeutic traditional information of plants of the Asteraceae family is kept alive through its transmission from generation to generation, especially among rural populations and indigenous communities, where herbal medicine remains a key pillar of health [13].

The present study focuses on three species of the Asteraceae family (*Solidago virgaurea*, *Tussilago farfara*, and *Helichrysum stoechas*) that are of significant phytochemical interest. Kraujalienė et al. [17] also subjected *S. virgaurea* leaves to pressurized liquid extraction (PLE) to recover polyphenolic compounds, confirming the effectiveness and green nature of the method, with the maximum yield and antioxidant capacity at 140 °C. Furthermore, the extracts improved the oxidative stability of rapeseed oil and emulsions in Oxipres and Rancimat tests, indicating their potential application in the food industry [17]. In contrast, for the other two species, the application of PLE remains limited, as most studies are based on conventional extraction techniques [5,18,19].

Response surface methodology (RSM) is a widely used statistical approach for the optimization of extraction processes, as it enables the simultaneous evaluation of multiple experimental factors and their interactions while reducing the number of experimental trials required [20]. Its ability to model complex interactions and identify optimal conditions has made it particularly valuable in extraction processes of bioactive compounds from plant matrices [21]. In parallel, partial least squares (PLS) analysis is a robust multivariate technique commonly used to handle highly collinear and complex datasets, allowing the extraction of meaningful correlations between chemical profiles and experimental factors [22]. Therefore, the combined use of RSM and PLS provides a comprehensive framework for both process optimization and data interpretation, enabling a deeper understanding of the factors influencing extraction efficiency and phytochemical profiles.

The present study introduces a comprehensive multiparametric approach to optimize PLE parameters in three plant species of the Asteraceae family, with incomplete or limited prior evaluation under PLE conditions. In contrast to previous studies that mainly focus on individual parameters or conventional extraction methods, this work combines experimental design and advanced multivariate analysis response surface methodology (RSM) to gain a deeper understanding of the relationships between extraction conditions and bioactivity. At the same time, the integration of Principal Component Analysis, multivariate correlation analysis, and Pareto plot analysis allows for simultaneous assessment of the contribution of factors and the chemical behavior of extracts, offering a more reliable and nonlinear interpretation framework compared to classical single-parameter approaches. Furthermore, the simultaneous comparison of PLE with conventional stirring extraction (STE) and the correlation with high-performance liquid chromatography (HPLC) profiles strengthens the possibility of linking process, chemical composition, and biological activity, highlighting PLE as not just a green but a targeted and selective extraction technology as well.

## 2. Materials and Methods

### 2.1. Chemicals and Reagents

For the needs of this specific study, the following reagents were used. Ethanol (≥99.8% *v*/*v*) from Panreac Co. (Barcelona, Spain), as well as sodium carbonate (≥99% *w*/*w*), 2,2-diphenyl-1-picrylhydrazyl (DPPH) (≥90% *w*/*w*), aluminum chloride (≥99% *w*/*w*), methanol (≥99.8% *v*/*v*), trichloroacetic acid (≥99% *w*/*w*), L-ascorbic acid (≥99% *w*/*w*), hydrochloric acid (37% *w*/*w*), and 2,4,6-tris(2-pyridyl)-*s*-triazine (TPTZ) (≥98% *w*/*w*), which were purchased from Sigma-Aldrich (Darmstadt, Germany). Iron (III) chloride (≥99% *w*/*w*) was acquired from Merck (Darmstadt, Germany). For the analysis of polyphenols by HPLC, high purity standards (≥99% *w*/*w*) were used, which were obtained from MetaSci (Toronto, ON, Canada). Deionized water was used in all experimental procedures, which was prepared through a deionization column system.

### 2.2. Instrumentation

The dried plant samples were ground with a Camry CR 4071 nutri pro professional blender (Adler Europe Group, Warsaw, Poland) to reduce the particle size and increase the contact surface during the extraction process. A pressurized liquid extraction (PLE) system from Fluid Management Systems Inc. (Watertown, MA, USA) and a heated magnetic stir plate from Heidolph Instruments GmbH & Co. KG (Schwabach, Germany) were used to perform the extractions. The extracts intended for temperature-controlled testing were placed in an Elmasonic P70H ultrasonic bath (Elma Schmidbauer GmbH, Singen, Germany). Spectrophotometric measurements were performed with a Shimadzu UV-1900i UV/Vis spectrophotometer (Kyoto, Japan). Finally, for the quantitative analysis of individual polyphenols, a Shimadzu CBM-20A high-performance liquid chromatography system equipped with an SPD-M20A diode array detector (Shimadzu Europa GmbH, Duisburg, Germany) was used. The separation of the compounds was performed on a Phenomenex Luna C18(2) column (100 Å, 5 μm, 4.6 mm × 250 mm) from Torrance (CA, USA), with the temperature maintained at 40 °C.

### 2.3. Dried Plant Leaves Material

For all experiments, dried leaves of three different plants were provided from a local store in Thessaloniki, Greece. From all three plant materials, the leaves were used, excluding flowering and fruiting tops. The dried leaves were ground into a fine powder, and the materials were stored at −40 °C until further analysis.

### 2.4. Experimental Design

A three-factor, three-level Box–Behnken response surface design was employed to investigate the influence of extraction parameters on the PLE process. The independent variables were solvent concentration $X_{1}$ (0, 50, 100% *v*/*v* ethanol), extraction temperature $X_{2}$ (40, 100, 160 °C), and extraction time $X_{3}$ (5, 15, 25 min). Each factor was evaluated at three coded levels (−1, 0, +1), generating a total of 15 experimental runs, including three replicated center points to estimate pure error. The coded and actual factor levels of the Box–Behnken design are presented in Table 1. A Box–Behnken design was selected because it offers high efficiency for estimating second-order effects while requiring fewer experimental runs than a full factorial design. The design strategy follows the classical RSM framework described by Box and Draper [23] and the original formulation of the Box–Behnken design [24].

The design matrix was constructed in JMP^®^ Pro 16 (SAS Institute Inc., Cary, NC, USA) using a second-order polynomial model structure. All linear terms, two-factor interactions, and quadratic terms were included in the model specification (Equation (1)):

(1) $$Y\;=\;\beta_{0}\;+\;\beta_{1}X_{1}\;+\;\beta_{2}X_{2}\;+\;\beta_{3}X_{3}\;+\;\beta_{12}X_{1}X_{2}\;+\;\beta_{13}X_{1}X_{3}\;+\;\beta_{23}X_{2}X_{3}\;+\;\beta_{11}X_{1}^{2}+\beta_{22}X_{2}^{2}\;+\;\beta_{33}X_{3}^{2}$$

This ensured that curvature and interaction effects among the extraction parameters could be adequately captured.

The run order was fully randomized to minimize systematic bias. Each experimental condition was extracted at least in duplicate, and all analytical determinations (TPC, FRAP, DPPH, AAC) were performed in triplicate. The same Box–Behnken design was applied independently to the three plant species (*Solidago virgaurea*, *Tussilago farfara*, *Helichrysum stoechas*) to allow direct comparison of parameter effects under identical experimental geometry.

No constraints were imposed on the factor space, and all extractions were performed under a constant pressure of 1700 psi and a fixed liquid-to-solid ratio of 20:1 mL/g, ensuring comparability between runs and between species.

### 2.5. Leaf Extraction

Dried leaf material from each plant species was extracted using two different techniques: conventional STE and PLE. The optimal values of liquid-to-solid ratio and ethanol concentration determined by PLE was also used in STE, ensuring a sounder comparison.

For the STE procedure, 1 g of dried plant material was mixed with 20 mL of solvent in 50 mL screw-cap glass bottles and extracted at 80 °C for 60 min on a heated magnetic stirrer set at 500 rpm. After extraction, the samples were centrifuged at 10,000× *g* for 10 min, and the supernatant was collected.

For the PLE experiments, the same solvent composition and liquid-to-solid ratio were applied. Extractions were carried out in a PLE system operated at a constant pressure of 1700 psi. The three extraction parameters—solvent concentration, temperature, and time—were varied according to the Box–Behnken experimental design described in Section 2.4. After each extraction cycle, the samples were centrifuged at 10,000× *g* for 10 min to separate the supernatant.

All extracts obtained from both techniques were stored at −40 °C until further analysis.

### 2.6. Spectrophotometric Evaluation of Total Polyphenolic Content (TPC)

Total polyphenol content (TPC) was determined spectrophotometrically by the Folin–Ciocalteu method, according to a previously published procedure [25]. Results were expressed as mg gallic acid equivalents (GAE) per g dry weight (dw). A calibration curve of gallic acid in aqueous solution was used for quantification, in the concentration range of 10–100 mg/L (R^2^ = 0.996), while measurements were performed at 740 nm with a Shimadzu UV-1900i UV/Vis spectrophotometer (Kyoto, Japan). Samples were incubated at 40 °C in an Elmasonic P70H ultrasonic bath from Elma Schmidbauer GmbH (Singen, Germany). All analyses were performed three times, and the average value of the measurements was used for the final evaluation. The TPC was determined and expressed as mg of gallic acid equivalents (GAE) per g of dry weight, using the following mathematical formula (Equation (2)):

(2) $$\mathrm{TPC}\;(\mathrm{mg}\;\mathrm{GAE}/g\;\mathrm{dw})\;=\;\frac{C_{\mathrm{TP}}\times V}{w}$$

where *w* denotes the dry mass of the sample (in g), whereas the total volume of the extraction medium is represented by *V* (in L).

### 2.7. Determination of Antioxidant Activity via Reducing Power (FRAP)

The antioxidant capacity of the extracts was determined according to a standard study, which describes in detail the electron transfer method [26]. The technique was based on measuring the reduction in iron from the +3 to +2 oxidation state by absorption at 620 nm. A calibration curve of ascorbic acid (50–500 μM in 0.05 M HCl solution, R^2^ = 0.9997) was used for quantification, while the results were expressed in μmol ascorbic acid equivalents (AAE) per g dry weight (dw). All analyses were performed on triplicate samples, and the mean value of the measurements was used for the final evaluation. The reducing power (*P*_R_) was calculated based on Equation (3) and expressed as μmol AAE per g dry weight:

(3) $$P_{R}\;(\mu \mathrm{mol}\;\mathrm{AAE}/g\;\mathrm{dw})\;=\;\frac{C_{AA}\times V}{w}$$

where *w* is defined as the mass of the dry sample in grams (g), while the parameter *V* corresponds to the total volume of the extractant (expressed in L).

### 2.8. Evaluation of the Free Radical Scavenging Activity

To determine the antiradical activity of the extracts, the previously described DPPH free radical scavenging method [26] was applied, which is based on the decrease in the absorbance of the solution due to neutralization of the stable DPPH radical by the antioxidant components of the samples. An ascorbic acid calibration curve (100–1000 μmol/L in methanol, R^2^ = 0.9926) was used to quantify the antiradical activity, while the results were expressed as μmol ascorbic acid equivalents (AAE) per g dry weight (dw). Each measurement was performed on three replicated samples, and the average value of the results was used for the final evaluation. The inhibition percentage (%) was calculated according to Equation (4), while the antiradical activity (*A*_AR_) was determined through the ascorbic acid calibration curve (*C*_AA_) according to Equation (5):

(4) $$\mathrm{Inhibition}\;\left(\%\right)\;=\;\frac{A_{515\left(i\right)}\;-\;A_{515\left(f\right)}}{A_{515\left(i\right)}}\times 100$$

(5) $$A_{\mathrm{AR}}\;\left(\mu \mathrm{mol}\;\mathrm{AAE}/g\;\mathrm{dw}\right)\;=\;\frac{C_{\mathrm{AA}}\times V}{w}$$

### 2.9. Determination of Ascorbic Acid Content (AAC)

The concentration of ascorbic acid in the examined samples was determined according to the method of Mantiniotou et al. [25] and expressed in mg per g dry weight. The procedure was based on the preparation of a reaction mixture in which the Folin–Ciocalteu reagent and trichloroacetic acid (TCA) were combined, aiming for the appropriate treatment of the extract before the spectrophotometric measurement. Specifically, 500 μL of Folin–Ciocalteu solution (10% *v*/*v*), 100 μL of sample extract, and 900 μL of 10% (*w*/*v*) trichloroacetic acid solution were added to Eppendorf tubes. The mixture was stirred and incubated for 10 min in dark conditions in order to prevent any photochemical alterations of the components. After completion of incubation, the absorbance was immediately measured at 760 nm via spectrophotometric analysis in order to determine the final ascorbic acid concentration.

### 2.10. Quantitative Determination of Polyphenolic Compounds by HPLC

Determination of the individual polyphenolic components of the extracts was performed by the HPLC method, in accordance with a previously published study by our research group [25]. The mobile phase consisted of a solution of 0.5% formic acid in acetonitrile (phase B) and 0.5% formic acid in aqueous phase (phase A). The gradient elution started from 0% of phase B and increased progressively to 40%, to 50% within 10 min, and then to 70% over the next 10 min, while this composition was kept constant for another 10 min. The flow rate of the mobile phase was kept constant at 1 mL/min. The identification of the compounds was based on the comparison of the retention time and absorption spectrum with those of pure compound standards. Quantitative analysis was performed through calibration curves of concentrations 0–50 μg/mL (Table A1). Detailed information on the identified polyphenolic compounds and their determination is provided in Appendix A.

### 2.11. Statistical Analysis

All statistical analyses were performed using JMP^®^ Pro 16 (SAS Institute Inc., Cary, NC, USA). The response surface methodology (RSM) models were fitted using the second-order polynomial structure described in Section 2.4, including all linear, interaction, and quadratic terms. Each experimental condition of the Box–Behnken design was extracted at least in duplicate, and all analytical measurements were conducted in triplicate; mean values were used for model fitting.

The Kolmogorov–Smirnov test was applied to assess the normality of the data. One-way analysis of variance (ANOVA), followed by Tukey’s HSD post hoc test, was used to evaluate differences among extraction conditions where appropriate. All results were expressed as mean values accompanied by their corresponding measures of variability.

Partial least squares (PLS) regression was performed in JMP^®^ Pro 16 to evaluate the multivariate relationships between extraction parameters and antioxidant responses. The X-block included the three coded factors (*X*_1_, *X*_2_, *X*_3_) and their quadratic/interaction terms, while the Y-block included TPC, FRAP, DPPH, and AAC. Model complexity was selected based on cross-validation and the minimum predicted residual sum of squares (PRESS).

Principal Component Analysis (PCA) and multivariate correlation analysis (MCA) were also conducted in JMP^®^ Pro 16. All responses were autoscaled prior to analysis. PCA was used to explore clustering patterns and shared variance among antioxidant assays, whereas MCA was used to compute pairwise correlations and generate hierarchical clustering heatmaps.

## 3. Results and Discussion

### 3.1. Extraction Optimization

The experimental data obtained from the Box–Behnken design were successfully fitted to second-order polynomial models for all responses (TPC, FRAP, DPPH, AAC) across the three Asteraceae species (*S. virgaurea*, *T. farfara*, *H. stoechas*), as presented in Table 2. The models exhibited high predictive power (Table 3), with R^2^ values ranging from 0.87 to 0.99 and adjusted R^2^ values above 0.91 for most responses. Lack-of-fit tests were non-significant (*p* > 0.05), confirming model adequacy.

In Table 4, all significant (*p* < 0.05) model terms are presented. Solvent concentration (*X*_1_) was consistently the most influential factor, showing strong negative linear and quadratic effects across all responses. This indicates that neither pure water nor absolute ethanol were ideal extraction media, while intermediate ratios (approximately 30–45% *v*/*v*) lead to the maximum extraction yield. Temperature (*X*_2_) had a significant positive effect, mainly in the species *S. virgaurea* and *H. stoechas*, suggesting that elevated temperatures (≥140 °C) facilitate the transport of compounds and the breakdown of plant structure. In contrast, extraction time (*X*_3_) appeared to have a limited effect, with the exception of FRAP in *H. stoechas*, where a significant nonlinear correlation was observed, indicating an optimal duration of approximately 15 min.

**Figure 1:**
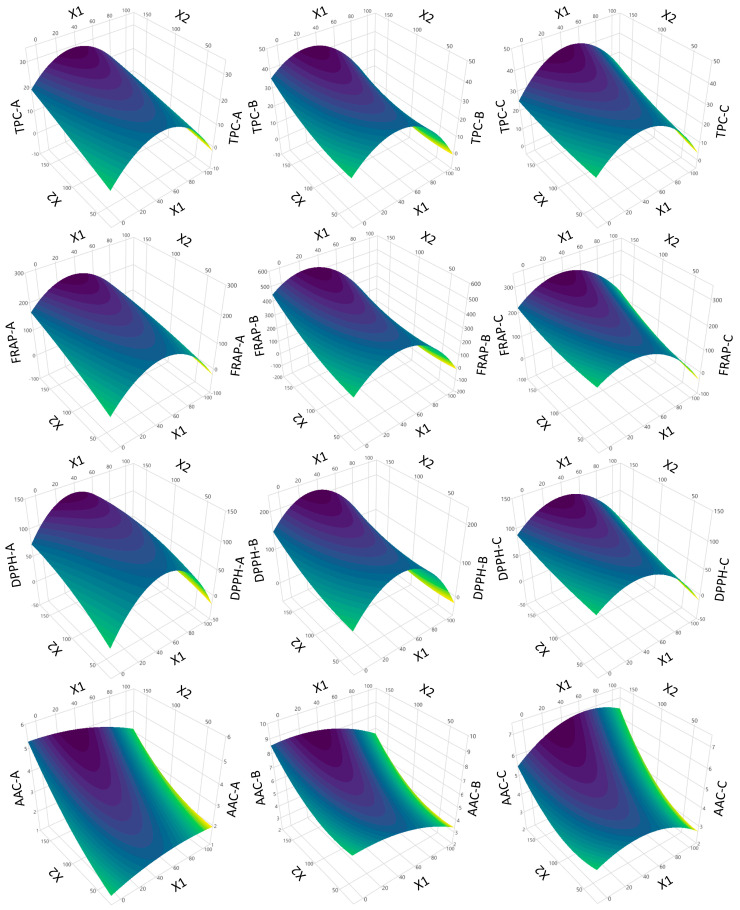
Three-dimensional response surface plots illustrating the combined effects of solvent concentration (*X*_1_) and temperature (*X*_2_) on the extraction performance of the three Asteraceae species: *Solidago virgaurea* (A), *Tussilago farfara* (B), and *Helichrysum stoechas* (C). The letters A–C refer to the three species and not to figure panels. Each row corresponds to a different antioxidant assay (TPC, FRAP, DPPH, and AAC), while the color gradients represent the magnitude of the response, with warmer colors indicating higher values. The plots highlight the curvature and interaction patterns captured by the RSM models and reveal the optimal extraction regions for each species and analytical response.

In Figure 1, the three-dimensional response surfaces graphically depict how temperature and solvent concentration interact, affecting the antioxidant activities of the three species. As can be seen in the graphs, the strong *X*_1_ effect is confirmed, as well as the positive linear trend of *X*_2_—findings that are perfectly aligned with ANOVA and PLS analyses. It is noteworthy that, in the species *H. stoechas* and *S. virgaurea*, the optima of the curves are much steeper. In contrast, *T. farfara* presents flatter surfaces, which suggests that it is less sensitive to modifications in extraction conditions. Finally, the differentiated morphology in the AAC diagrams reinforces the conclusion of the multivariate analysis: the AAC index moves in a different way compared to TPC, FRAP, and DPPH.

### 3.2. Impact of Extraction Parameters to Assays Through Pareto Plot Analysis

**Figure 2:**
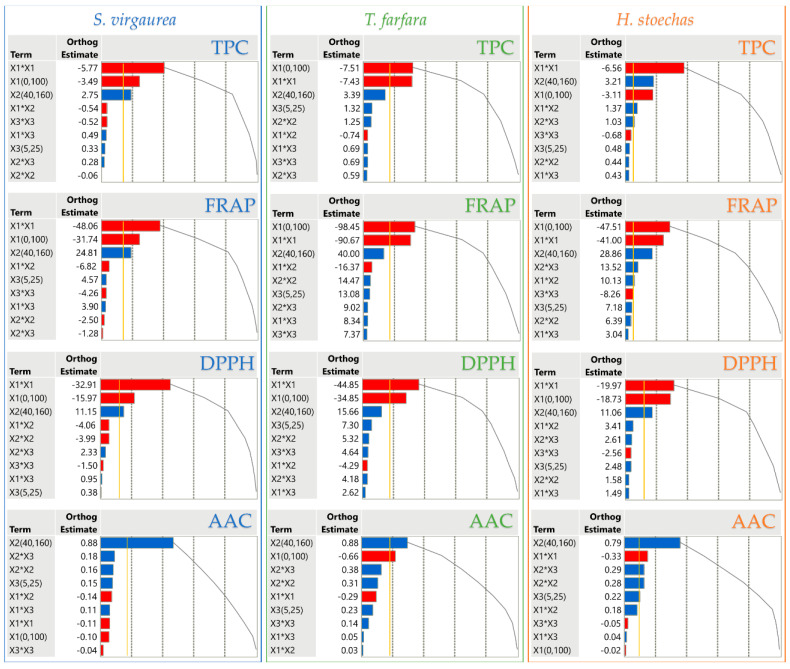
Pareto charts of standardized effects (TPC, FRAP, DPPH, and AAC for *S. virgaurea*, *T. farfara*, and *H. stoechas*).

Pareto plots (Figure 2) revealed that *X*_1_ and *X*_1_^2^ were the dominant contributors to variation in TPC, FRAP, and DPPH across all species. In *H. stoechas*, *X*_2_ and its interactions (*X*_1_ × *X*_2_; *X*_2_ × *X*_3_) also emerged as significant, highlighting the synergistic effect of temperature and solvent polarity. For *T. farfara*, the extraction was primarily governed by solvent concentration, with minimal influence from temperature or time.

The strong curvature observed for *X*_1_ suggests a narrow optimal range, where excessive ethanol content may reduce solubility of moderately polar phenolics [27,28,29], while excessive water may limit matrix penetration [30]. The positive effect of temperature aligns with PLE principles, where elevated thermal energy enhances mass transfer and reduces solvent viscosity [31].

### 3.3. Analysis of the Extracts

Among the three species, *H. stoechas* consistently exhibited higher FRAP and DPPH values, indicating a stronger radical scavenging capacity under the tested extraction conditions. Notably, although *S. virgaurea* presented the highest TPC, its total reducing power was lower than that of *H. stoechas*, suggesting that qualitative phytochemical composition and matrix effects influence the activity more than simple quantitative concentration. In contrast, the more modest performance of *T. farfara* may reflect either an inherently lower phenolic load or a suboptimal release of bioactive compounds during the PLE process. Due to these strong characteristics, the extracts of *H. stoechas* and *S. virgaurea* may emerge as highly promising for the development of advanced parapharmaceutical formulations.

### 3.4. Principal Component Analysis (PCA) and Multivariate Correlation Analysis (MCA)

**Figure 3:**
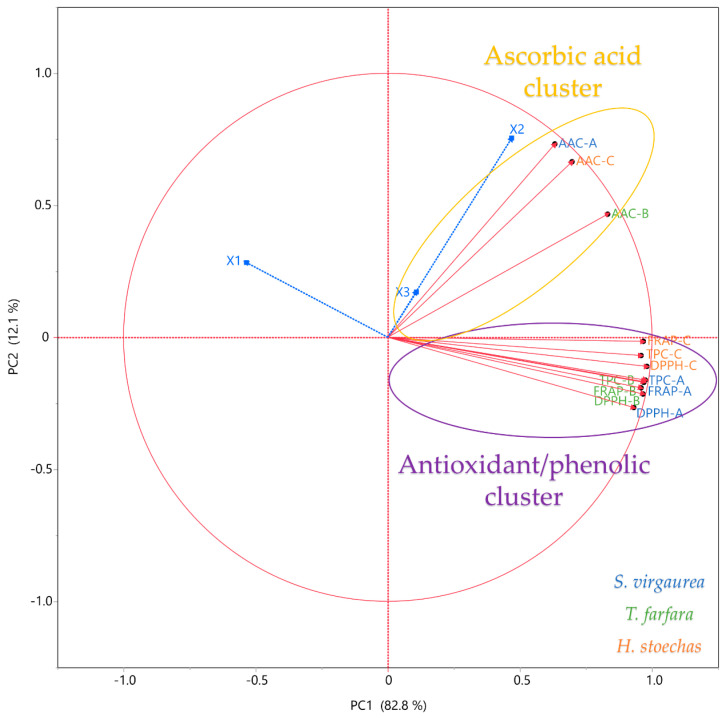
Principal Component Analysis (PCA) biplot illustrating the distribution of antioxidant responses and ascorbic acid content across the three Asteraceae species: *S. virgaurea* (blue), *T. farfara* (green), and *H. stoechas* (orange). The first two principal components (PC1 = 82.8%, PC2 = 12.1%) explain 94.9% of the total variance. Red vectors represent the contribution of each response variable, with TPC, FRAP, and DPPH clustering along PC1, and AAC variables aligning with PC2. The plot reveals two distinct clusters, an antioxidant/phenolic cluster and an ascorbic acid cluster, confirming the multivariate separation of response types. Dashed blue vectors indicate the influence of extraction parameters (*X*_1_, *X*_2_, *X*_3_) on sample positioning.

As seen in Figure 3, Principal Component Analysis (PCA) demonstrated a highly coherent structure among the antioxidant responses of the three Asteraceae species. The first principal component (PC1) exhibited an eigenvalue of 9.94 and accounted for 82.84% of the total variance, while PC2 explained an additional 12.07%, bringing the cumulative variance to 94.91%. This indicates that almost all variability in the dataset can be summarized in a two-dimensional space.

Loadings showed that TPC, FRAP, and DPPH from all three species loaded very strongly and positively on PC1 (0.93–0.98), confirming that these assays describe a common underlying antioxidant/phenolic axis. In contrast, AAC values loaded primarily on PC2 (0.66–0.73), indicating that total antioxidant capacity captures a different biochemical dimension compared to the phenolic-driven assays.

**Figure 4:**
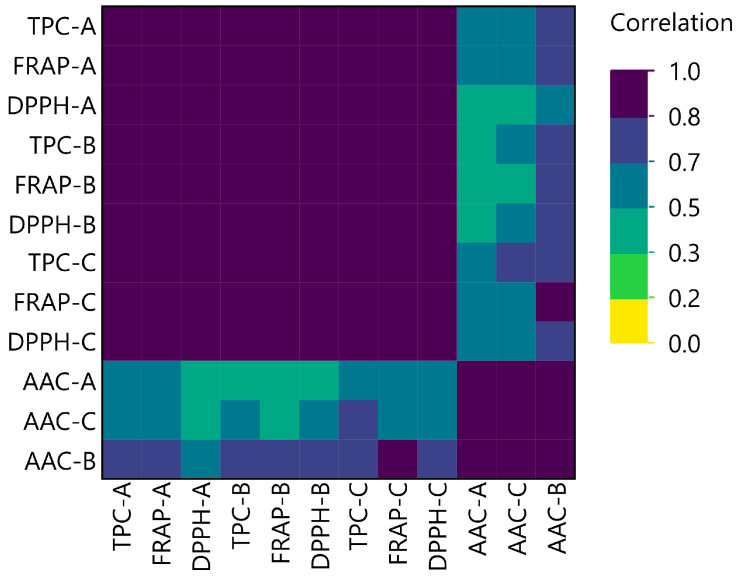
Multivariate correlation heatmap showing pairwise Pearson correlations between antioxidant responses and ascorbic acid content across all species. Strong positive correlations are represented by darker purple tones, while weaker or negative correlations appear in yellow. The clustering of TPC, FRAP, and DPPH responses in the purple region confirms their high interdependence, whereas AAC responses show lower correlations, indicating a distinct antioxidant mechanism. The heatmap supports the PCA-based separation between antioxidant/phenolic-driven and ascorbic-acid-driven responses.

Multivariate correlation analysis (MCA, Figure 4) supported these findings. TPC, FRAP and DPPH exhibited very strong positive correlations across all species (r = 0.90–0.99), while AAC showed moderate correlations with the other assays (r = 0.45–0.89), reinforcing its complementary nature. The correlation heatmap and clustering analysis further revealed two distinct groups: (1) a large cluster containing all TPC, FRAP, and DPPH variables (cluster R^2^ = 0.95); and (2) a smaller cluster containing all AAC variables (cluster R^2^ = 0.91). This separation confirms that AAC reflects a broader antioxidant mechanism beyond phenolic content alone.

The PCA score plots showed that samples extracted at intermediate solvent concentrations and high temperatures tended to cluster together, consistent with the RSM-derived optimal conditions. Moreover, *H. stoechas* formed a partially distinct group in PCA space, reflecting its unique phytochemical profile and the stronger sensitivity of its antioxidant responses to extraction parameters.

The statistical differentiation of AAC from the TPC-FRAP-DPPH group in the double PCA plot (Figure 3) is one of the most important qualitative findings of the study. Although antioxidant assays are usually correlated with the phenolic profile, the deviation of AAC suggests the activation of specific chemical compounds under the intense PLE conditions (160 °C). Three main axes explain this phenomenon. First, the nature of Asteraceae species, which are rich in organic acids and structural polysaccharides, favors the hydrolytic release of non-phenolic components due to the high thermal energy and pressure (1700 psi). These components are specifically detected by the AAC assay. In addition, Maillard reactions activated at 160 °C through the interaction of sugars and amino acids, lead to the formation of melanoidins. These compounds have excellent reducing power, significantly increasing AAC values. Finally, the nonlinear bioactivity that characterizes pressurized systems must be taken into account. As previously reported by Athanasiadis et al. [5] and Mantiniotou et al. [32], PLE parameters—particularly high temperatures—trigger complex bioactive shifts.

In conclusion, PCA separation confirms that optimized PLE does not simply increase the quantity but qualitatively transforms the extract, giving it a unique chemical identity with enhanced total antioxidant capacity.

### 3.5. Partial Least Squares (PLS) Analysis

Partial least squares regression was employed to complement the RSM findings and assess the multivariate predictive power of the extraction parameters on all antioxidant responses (TPC, FRAP, DPPH, AAC) across the three Asteraceae species. The NIPALS algorithm, with nine latent factors, explained 100% of the variation in X and 99.999% in Y, with a cumulative Q^2^ = 0.986, indicating excellent predictive performance. The minimum root mean PRESS was 0.256, while van der Voet’s test confirmed model validity (*p* = 1.000).

**Figure 5:**
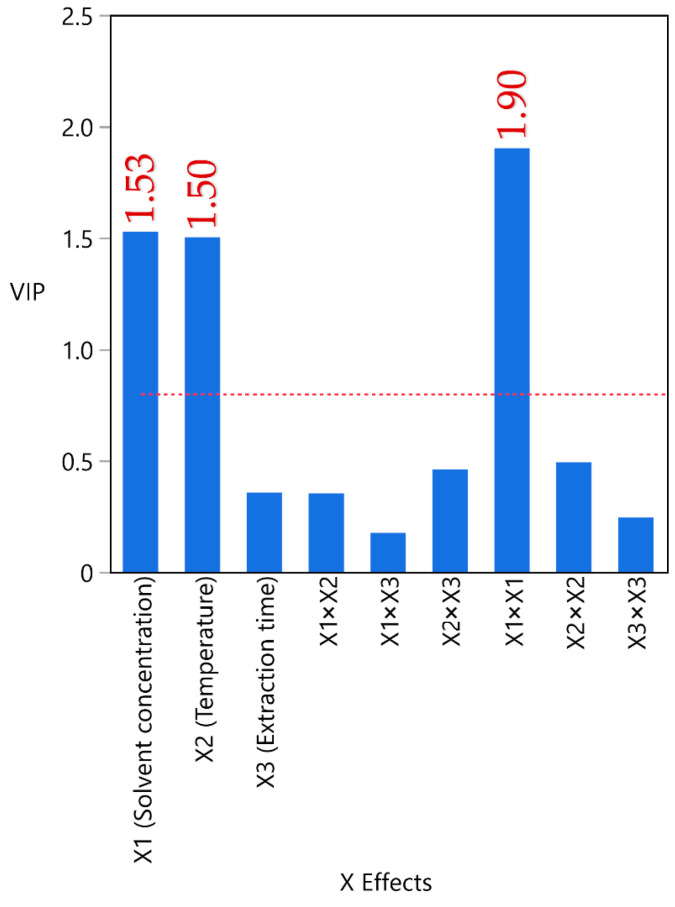
Variable Importance in Projection (VIP) scores derived from the PLS regression model, indicating the relative contribution of each factor and interaction to the prediction of antioxidant responses (TPC, FRAP, DPPH, AAC) across all species. The red dotted line marks the threshold of VIP = 0.8, above which variables are considered highly influential.

The Variable Importance in Projection (VIP) scores (Figure 5) identified solvent concentration (*X*_1_), temperature (*X*_2_), and the quadratic term *X*_1_^2^ as the most influential predictors, with VIP values of 1.53, 1.50, and 1.90, respectively. These findings align with the RSM models and confirm that extraction efficiency is primarily governed by solvent polarity and thermal energy. Time-related and interaction terms (*X*_3_; *X*_1_ × *X*_2_; *X*_2_ × *X*_3_; *X*_3_^2^) exhibited lower VIP scores (<0.5), indicating limited contribution to the overall model.

**Figure 6:**
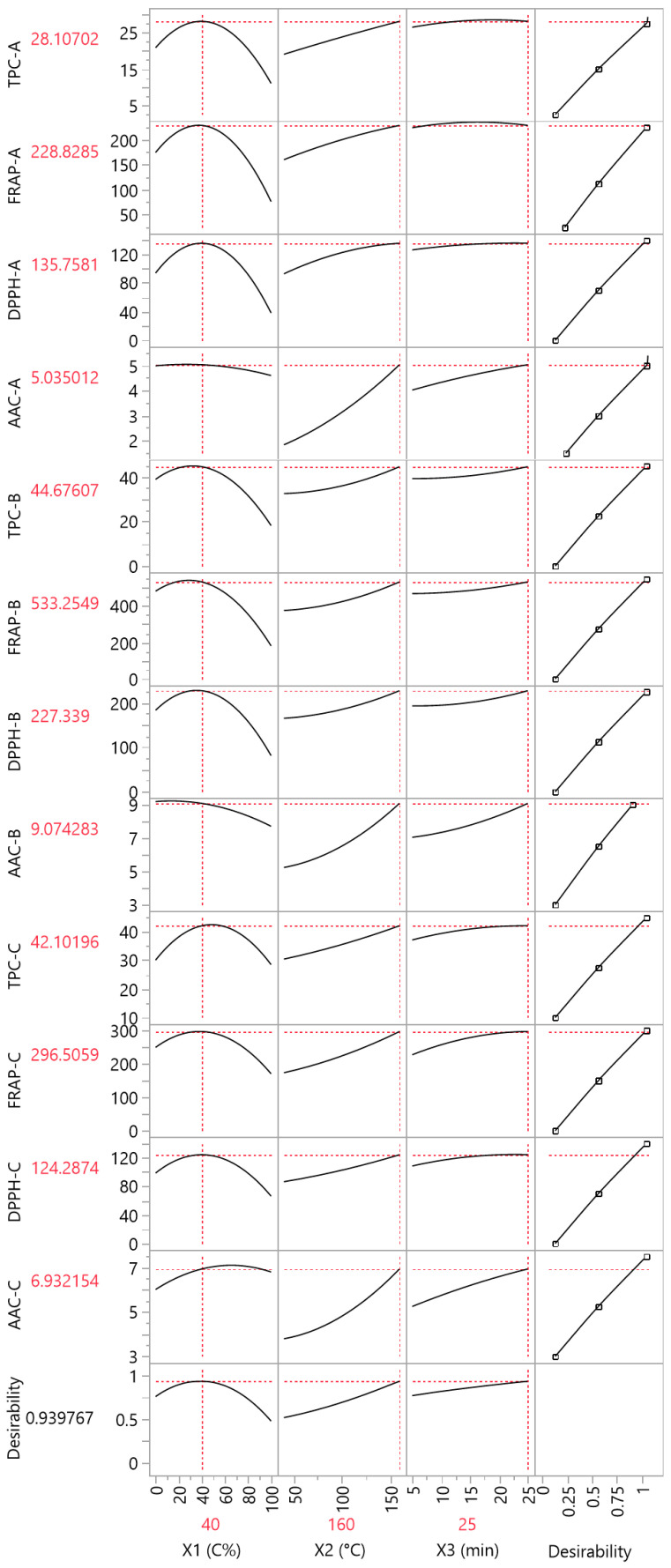
Prediction profiler illustrating the individual and combined effects of solvent concentration (*X*_1_), temperature (*X*_2_), and extraction time (*X*_3_) on the predicted values of antioxidant responses (TPC, FRAP, DPPH, AAC) across all species. The red dotted lines indicate the optimal levels for each factor (*X*_1_ = 40% *v*/*v*, *X*_2_ = 160 °C, *X*_3_ = 25 min), while the red numbers on the left denote the predicted maxima for each response. The final panel shows the composite desirability function, with an overall score of 0.94, confirming that the selected conditions simultaneously optimize all responses within the experimental domain.

The prediction profiler (Figure 6) further revealed that optimal extraction conditions for maximizing all responses simultaneously were *X*_1_ = 40% *v*/*v*, *X*_2_ = 160 °C, and *X*_3_ = 25 min, yielding a composite desirability score of 0.94. These values are consistent with the RSM-derived optima and reinforce the robustness of the multivariate approach.

### 3.6. Comparison of Optimized PLE Conditions with STE

To assess the practical advantages of PLE, the optimized PLE conditions obtained from the RSM–PLS workflow were compared with a conventional magnetic STE. The STE protocol was performed using a solid-to-solvent ratio of 1 g/20 mL, 40% ethanol, 80 °C, 60 min extraction time, and 500 rpm agitation. In contrast, the optimized PLE method employed the same solid-to-solvent ratio and solvent composition but operated at 160 °C for 25 min under 1700 psi. Despite using identical solvent polarities, the two methods differ substantially in mass transfer mechanisms, thermal energy inputs, and extraction kinetics.

Across all species and antioxidant assays, PLE generally produced equal or superior extraction performance relative to STE; however, several responses showed statistically significant differences that highlight the strengths of each method. For *S. virgaurea* (A), PLE produced a significantly higher AAC-A value compared to STE (5.84 vs. 3.74 mg/g, *p* < 0.05), indicating enhanced extraction of compounds contributing to total antioxidant capacity. TPC-A and FRAP-A did not differ significantly between methods, while DPPH-A was significantly lower under PLE (69.18 vs. 82.16 μmol AAE/g, *p* < 0.05), suggesting that some radical scavenging constituents may be more efficiently extracted under the milder STE conditions. For *T. farfara* (B), PLE yielded significantly higher AAC-B (9.15 vs. 7.80 mg AA/g, *p* < 0.05), confirming improved extraction of total antioxidant capacity components. In contrast, TPC-B and DPPH-B were significantly higher under STE (57.91 vs. 52.19 mg GAE/g and 280.80 vs. 246.86 μmol AAE/g, respectively; *p* < 0.05), indicating that certain phenolics and radical scavenging compounds may be more sensitive to the harsher PLE conditions. For *H. stoechas* (C), PLE again produced significantly higher AAC-C (7.92 vs. 5.33 mg/g, *p* < 0.05), while TPC-C was significantly higher under STE (47.32 vs. 43.93 mg GAE/g, *p* < 0.05). FRAP-C and DPPH-C showed no significant differences between methods.

The comparison between PLE and STE (Table 5) highlights the distinct extraction kinetics of the pressurized systems and focuses on the efficient recovery of phenolic compounds. A key finding of this study is the comparison of TPC in the three species, where the methods showed varying efficiencies. It is important to emphasize that there is currently limited literature on the optimization of TPC recovery via pressure extraction for these specific Asteraceae species, which highlights the novelty and necessity of the present study.

For *S. virgaurea* (A), the PLE method achieved a TPC of 32.03 mg GAE/g dw, a value significantly higher than those reported in the literature for studies applying conventional or other alternative techniques. In particular, Yavorska and Vorobets [33], when studying extracts of the related species *Solidago canadensis* using aqueous and hydroethanolic solvents, reported TPC values ranging from 3.54 to 17.36 mg GAE/g dw, depending on the plant part (up to 17.36 mg GAE/g dw).

Furthermore, recent data from Abdel-Gawad et al. [34], who investigated the effect of nano-fertilization on the biological potential of *S. virgaurea*, demonstrated that although new cultivation techniques can enhance secondary metabolism, TPC concentrations remained at lower levels than those achieved in the present work. These results demonstrate that, while the quality of the raw material is critical, the extraction method is the determining factor for maximizing yield. Our findings suggest that for *S. virgaurea*, PLE is a reliable high-yield alternative for phenolic isolation, filling a notable gap in the current literature.

Regarding *T. farfara* (B), despite its long-standing medicinal use, traditional studies on its optimized extraction under pressure conditions are almost non-existent. While Horozić et al. [9] provide baseline TPC values using conventional methods, it is worth noting that their protocol requires an extended extraction period of 20 h to reach equilibrium. In stark contrast, our optimized PLE achieves comparable TPC (52.19 mg GAE/g) in just 25 min. While STE showed slightly higher yields (57.91 mg GAE/g), suggesting a possible thermal sensitivity of some phenolic compounds that has not yet been documented in the limited literature, this minimal loss is largely compensated for by the drastic reduction in extraction time by 48-fold, establishing PLE as a superior green alternative.

Regarding the species *H. stoechas* (C), the optimized PLE method yielded 43.93 mg GAE/g dw, a value that highlights the impressive superiority over recent literature data. Specifically, Marques et al. [35], when applying conventional hydroalcoholic extraction, reported a TPC equal to 119.6 mg GAE/g extract. However, when this value is normalized to the initial dry weight of the plant and takes into account the low extraction yield of 7.13% that was recorded, the total recovery corresponds to only 8.53 mg GAE/g dw. This comparison reveals that the developed PLE approach achieved a five-fold increase (500%) in the recovery of phenolic components, simultaneously breaking the “time barrier” by achieving an impressive 80% reduction in extraction time (25 min vs. 120 min required by the conventional method). This dramatic improvement suggests that the synergy of high pressure (1700 psi) and temperature (160 °C) causes an effective disruption of the hard lignocellulosic matrix of the *H. stoechas*, thereby “unlocking” bound phenolic fractions that remain intact during simple stirring. Finally, the superiority of PLE is not limited to quantity but also extends to quality, as the much higher AAC values (7.92 vs. 5.33 mg/g dw) demonstrate that the pressurized system selectively isolates the most potent antioxidant molecules, making PLE the most promising “green” technology for high-performance industrial applications.

Overall, the consistently elevated AAC values under PLE across all species demonstrate the strong ability of high-temperature PLE to enhance the recovery of compounds contributing to total antioxidant capacity. Conversely, STE outperformed PLE in several TPC and DPPH measurements, suggesting that some phenolic and radical scavenging constituents may be partially degraded or structurally altered under the more intense PLE conditions. These findings highlight that while PLE is highly effective for maximizing total antioxidant capacity, STE may preserve specific phenolic subclasses more efficiently.

The comparison between the RSM-derived optimal extraction conditions and the PLS regression predictions demonstrated excellent agreement (r = 0.9889, *p* < 0.0001). The linear fit between the predicted responses and the PLS regression output yielded R^2^ = 0.9779, confirming that the multivariate PLS model fully supports the optimal conditions identified by RSM. These results indicate that the extraction optimum (40% solvent, 160 °C, 25 min) is robust, stable, and consistent across both modeling approaches. The strong concordance between experimental PLE performance, RSM optimization and PLS predictive modeling further validates the superiority of PLE over conventional STE, both in extraction efficiency and methodological reliability [32].

### 3.7. Polyphenolic Profiling by HPLC

**Figure 7:**
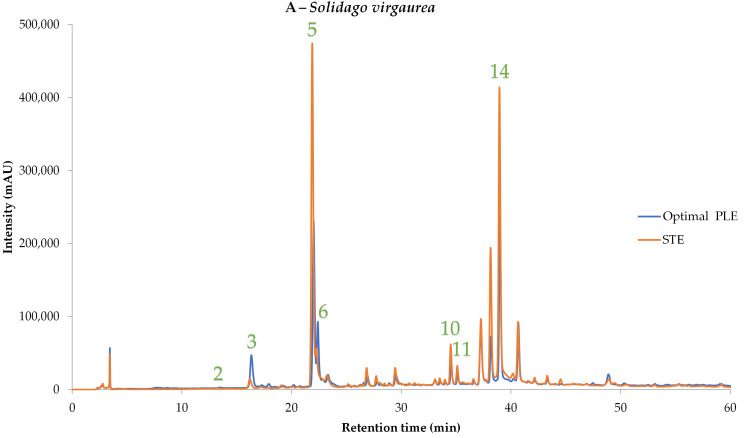

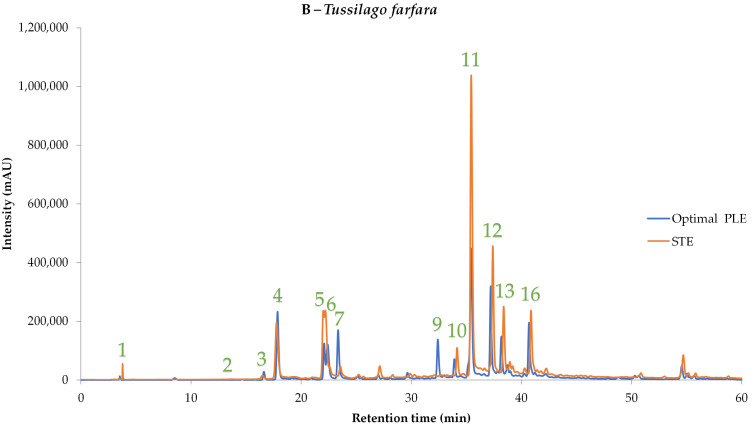

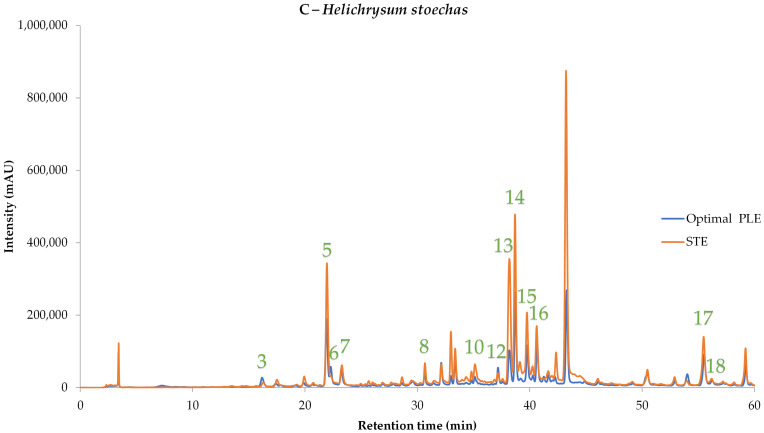
HPLC chromatograms at 320 nm comparing the polyphenolic profiles of *Solidago virgaurea* (**A**), *Tussilago farfara* (**B**), and *Helichrysum stoechas* (**C**) extracted via optimized pressurized liquid extraction (PLE, blue) and conventional stirring extraction (STE, orange). Peak intensities and retention times reveal distinct differences in compound abundance and composition between methods. The numbered peaks correspond to the compound identities listed in Table 6.

HPLC was employed to characterize and quantify the major polyphenolic constituents extracted from *S. virgaurea*, *T. farfara*, and *H. stoechas* using both optimized PLE and conventional STE. The chromatographic profiles (Figure 7) revealed substantial qualitative and quantitative differences between the two extraction methods, reflecting the influence of temperature, pressure, and extraction kinetics on the recovery of individual phenolic compounds.

Across all species, STE yielded a higher total concentration of identified polyphenols compared to PLE (Table 6). This trend was particularly pronounced in *T. farfara* (14.45 vs. 9.02 mg/g) and *H. stoechas* (9.01 vs. 5.78 mg/g), indicating that the milder thermal conditions and longer extraction time of STE favor the preservation and solubilization of a broader range of phenolic constituents. In *S. virgaurea*, STE also produced a higher total phenolic yield (6.39 vs. 4.45 mg/g). In Table 6, all results from HPLC-DAD are presented, while in Table A1, the equations of calibration curves of all identified compounds, along with their R^2^ values, LOD and LOQs are provided.

Regarding the species *S. virgaurea* (A), HPLC-DAD analysis identified chlorogenic acid (1.19 mg/g dw) as the dominant bioactive marker. Due to the limited literature on the PLE of this species, the results were compared with the closely related subspecies *S. virgaurea* subsp. *gigantea*. Although taxonomic differences and geographical origin justify the deviations in absolute concentrations, this comparison is crucial for the validation of the main chemical tracers of the genus Solidago. The most striking finding lies in the technological efficiency, as while Hwang et al. [36] required an exhaustive conventional extraction of 7 h (420 min) to recover polyphenols, our optimized PLE approach achieved a comparable and scientifically valid phytochemical footprint in just 25 min. This 16-fold reduction in operational time, without any compromise in the qualitative integrity of the extract, highlights PLE as a superior and industrially competitive technology, capable of “unlocking” the bioactive potential of Asteraceae in the minimum possible time. A critical finding of this study is the analytical selectivity of PLE. Specifically, in *T. farfara*, the caffeoyl quinic acid derivative 4 (0.66 mg/g dw) was detected exclusively in the PLE extracts and remained undetected (n.d.) under conventional STE conditions. This suggests that the combination of high pressure (1700 psi) and temperature (160 °C) effectively disrupts the rigid lignocellulosic matrix, ‘unlocking’ specific phenolic fractions that are inaccessible to simple stirring extraction. This transformative efficiency and chemical diversity were equally evident for *T. farfara* and *H. stoechas*, as confirmed by our comparative analysis. The polyphenolic profiling of the optimized PLE extracts via HPLC-DAD revealed a sophisticated chemical fingerprint, which is in high agreement with the recent comprehensive mapping of Balkan Asteraceae by Vojvodić et al. [18]. While Vojvodić et al. [18] established the baseline bioactive potential of *S. virgaurea* and *T. farfara* using a 24 h conventional extraction, the present study introduces a significant technological leap our optimized PLE protocol achieved a comparable, if not superior, recovery of key hydroxycinnamic acids and flavonoids in just 25 min. This nearly 57-fold reduction in extraction time, without compromising the integrity of thermolabile compounds, highlights PLE as a transformative green technology. The pharmaceutical relevance of these recovered metabolites, particularly the caffeoylquinic acid derivatives, is further validated by the findings of Wu et al. [7], who identified these specific compounds as the primary drivers of the antitussive and anti-inflammatory activities of *T. farfara*. By bridging the gap between cutting-edge extraction efficiency and proven pharmacological efficacy, our results demonstrate that PLE is not merely an alternative method, but a superior tool for the rapid standardization of high-potency therapeutic extracts from these underutilized species. The qualitative and quantitative analysis of the identified polyphenolic compounds further underscores the strategic advantage of PLE. In all three species, the extracts were dominated by hydroxycinnamic acids (mainly chlorogenic and neochlorogenic acid isomers) and flavonoid glycosides, such as rutin and kaempferol derivatives. This observation is in line with the recent study by Athanasiadis et al. [37], which argued that the synergy of these metabolites forms the potent antioxidant base of Mediterranean Asteraceae.

Building upon this methodological foundation, the present study introduces PLE as a significant evolutionary step. While previous work has relied on conventional stirring and ultrasound-assisted extraction for the recovery of phenolic constituents [18], our optimized PLE protocol achieved a comparable chemical profile within only 25 min, highlighting the efficiency of high-temperature, high-pressure green extraction technologies. Notably, the elevated thermal conditions of PLE facilitated the partial hydrolysis of matrix-bound phenolics, promoting the release of both glycosylated derivatives and aglycones, such as apigenin and kaempferol, which is in agreement with recent findings reported by Vojvodić et al. [18]. Furthermore, the identification of these compounds validates the stability of the plant’s phytochemical signature under subcritical conditions, confirming that PLE does not cause thermal degradation of these key bioactive markers, but rather maximizes their extraction kinetics, establishing it as the most competitive method for industrial food and pharmaceutical applications.

Despite the lower total yield, PLE selectively enhanced the extraction of specific compounds. Neochlorogenic acid was significantly higher under PLE in all three species, suggesting that elevated temperature and pressure facilitate the release of certain caffeoylquinic acid isomers from plant matrices. Similarly, PLE produced higher levels of apigenin derivatives in *T. farfara* and *H. stoechas*, indicating that these flavonoids may be more efficiently liberated under subcritical hydroalcoholic conditions.

Conversely, STE consistently yielded higher concentrations of chlorogenic acid, kaempferol-3-glucoside and several caffeoylquinic acid derivatives, particularly in *T. farfara* and *H. stoechas*. These compounds are known to be thermolabile or susceptible to structural transformation at elevated temperatures, which may explain their reduced abundance under PLE. The significantly higher levels of apigenin-7-*O*-glucoside and kaempferol in STE extracts further support the notion that prolonged extraction at moderate temperatures preserves glycosylated flavonoids more effectively.

Overall, the HPLC data demonstrate that PLE and STE produce extracts with distinct polyphenolic fingerprints. PLE favors the extraction of specific hydroxycinnamic acids and aglycone-rich flavonoids, whereas STE yields a broader and more abundant phenolic profile dominated by chlorogenic acid derivatives and glycosylated flavonoids. These compositional differences are consistent with the antioxidant assay results, where PLE systematically increased the AAC, while STE often produced higher TPC and DPPH values, reflecting its greater efficiency in extracting glycosylated and chlorogenic-acid-rich phenolics. The combined findings highlight that extraction technology not only affects total phenolic yield but also shapes the qualitative chemical composition of the extracts, ultimately influencing the antioxidant behavior of each species.

## 4. Conclusions

The present study highlighted the difference between PLE and conventional STE in three species of the Asteraceae family (*Solidago virgaurea*, *Tussilago farfara*, and *Helichrysum stoechas*). The application of PLE demonstrated significant superiority in the extraction of bioactive polyphenolic compounds and in maximizing the AAC, compared to the traditional STE. The optimal extraction condition with PLE (40% *v*/*v* ethanol, 160 °C, 25 min, 1700 psi) was confirmed both experimentally and through RSM and PLS models, which ensured high predictive accuracy and reliability of the results. A major finding of this research is the analytical selectivity and molecular transformation induced by subcritical conditions. HPLC analysis revealed that PLE achieves selective extraction of compounds such as neochlorogenic and apigenin derivatives; notably, PLE successfully ‘unlocked’ bioactive fractions that remained undetected (n.d.) under conventional stirring, such as specific caffeoylquinic acid derivatives in *T. farfara*. Furthermore, the observed shift in concentrations indicates that the high thermal energy of PLE (160 °C) promotes beneficial thermal isomerization, transforming chlorogenic acid into its neochlorogenic isomer, thereby yielding extracts with a unique chemical ‘signature’.

While STE maintained higher values of TPC and DPPH activity in some species, indicating that certain heat-sensitive flavonoid glycosides are better preserved under milder conditions, this minimal loss is compensated by the markedly shorter extraction time of PLE. In the present study, PLE required only 25 min compared to 60 min for STE (2.4-fold reduction), while the reported literature for conventional extraction of Asteraceae species often exceed several hours, leading to reductions of up to 50–60-fold under comparable solvent systems. This distinction has now been clarified in the manuscript.

## Figures and Tables

**Table 1:** Box–Behnken experimental design and coded factor levels, along with their actual values.

Independent Variables	Coded Units	Coded Levels		
		−1	0	1
Solvent concentration (*C*, % *v*/*v*)	*X*_1_	0	50	100
Extraction temperature (*T*, °C)	*X*_2_	40	100	160
Extraction time (*t*, min)	*X*_3_	5	15	25

**Table 2:** Design points and corresponding actual values for the three‑extract Box–Behnken experiment.

Design Point	Coded Factor Levels			Actual (*S. virgaurea*)				Actual (*T. farfara*)				Actual (*H. stoechas*)			
	*X*_1_	*X*_2_	*X*_3_	TPC-A	FRAP-A	DPPHA	AAC-A	TPC-B	FRAPB	DPPH-B	AAC-B	TPC-C	FRAP-C	DPPH-C	AAC-C
1	−1	1	0	22.68	195.82	96.48	4.94	39.45	489.24	171.94	8.32	30.80	237.83	97.14	5.29
2	0	−1	1	20.42	171.97	99.74	2.28	36.67	423.38	183.57	5.85	31.10	171.98	85.08	3.90
3	0	1	−1	24.11	203.25	116.30	3.53	32.23	382.05	162.56	6.10	35.78	208.40	102.94	5.18
4	0	0	0	22.79	194.00	115.86	2.89	28.64	333.44	147.46	5.33	35.02	221.65	100.67	4.56
5	−1	0	1	15.58	136.77	80.12	2.85	26.50	315.30	121.48	6.14	24.80	187.05	78.45	3.92
6	1	0	1	7.37	60.55	30.70	2.85	9.65	94.31	37.81	4.65	18.47	63.83	32.84	4.07
7	1	1	0	11.53	86.88	46.35	4.27	14.97	139.90	58.30	6.53	27.06	151.82	59.13	5.87
8	−1	0	−1	17.24	141.50	81.03	3.05	29.74	360.82	135.01	6.63	26.12	192.42	85.11	3.87
9	0	0	0	24.87	200.30	126.16	3.10	38.12	450.66	192.90	6.55	36.28	228.17	99.05	4.61
10	0	0	0	24.53	203.96	129.46	3.05	28.02	341.21	140.86	5.42	36.75	214.54	104.62	4.74
11	−1	−1	0	10.79	79.46	35.52	1.42	21.39	250.30	91.87	5.42	25.47	188.36	82.16	3.96
12	0	−1	−1	19.91	152.38	109.47	2.37	31.18	373.43	156.44	5.76	32.83	191.37	80.70	3.99
13	1	−1	0	3.85	23.34	16.85	1.83	2.61	27.76	11.45	3.36	11.11	23.88	17.70	3.13
14	1	0	−1	5.27	35.04	24.26	2.17	7.56	75.25	31.02	4.78	16.45	45.69	27.98	3.74
15	0	1	1	26.82	212.95	124.64	4.81	42.31	501.85	222.09	9.10	42.03	293.71	127.55	7.31

Note: A = *Solidago virgaurea*, B = *Tussilago farfara*, C = *Helichrysum stoechas*.

**Table 3:** Model adequacy statistics for all responses (RSM).

Species	Response	R^2^	Adj R^2^	RMSE	Lack-of-Fit *p*
*S. virgaurea*	TPC	0.983	0.952	1.68	0.25
	FRAP	0.983	0.952	14.61	0.25
	DPPH	0.969	0.914	11.94	0.17
*T. farfara*	TPC	0.918	0.771	5.86	0.50
	FRAP	0.931	0.807	67.3	0.51
	DPPH	0.939	0.828	26.64	0.59
	AAC	0.875	0.649	0.83	0.37
*H. stoechas*	TPC	0.989	0.97	1.47	0.21
	FRAP	0.991	0.976	11.66	0.20
	DPPH	0.99	0.971	5.36	0.17

**Table 4:** Significant model terms (*p* < 0.05).

Species	Response	Significant Terms
*S. virgaurea*	TPC	*X*_1_, *X*_2_, *X*_1_^2^
	FRAP	*X*_1_, *X*_2_, *X*_1_^2^
	DPPH	*X*_1_, *X*_2_, *X*_1_^2^
*T. farfara*	TPC	*X*_1_, *X*_1_^2^
	FRAP	*X*_1_, *X*_1_^2^
	DPPH	*X*_1_, *X*_1_^2^
	AAC	*X*_1_, *X*_2_
*H. stoechas*	TPC	*X*_1_, *X*_2_, *X*_1_^2^, *X*_1_ × *X*_2_, *X*_2_ × *X*_3_
	FRAP	*X*_1_, *X*_2_, *X*_1_^2^, *X*_3_^2^, *X*_1_ × *X*_2_, *X*_2_ × *X*_3_
	DPPH	*X*_1_, *X*_2_, *X*_1_^2^

**Table 5:** Comparison of predicted PLS regression values, optimized PLE results, and conventional stirring extraction (STE) for all antioxidant responses across the three Asteraceae species.

Responses	PLS Regression	Optimal PLE	STE
TPC-A	28.11	32.03 ± 1.12	31.69 ± 1.07
FRAP-A	228.83	274.22 ± 24.64	282.58 ± 13.23
DPPH-A	135.76	69.18 ± 4.23	82.16 ± 3.62 *
AAC-A	5.04	5.84 ± 0.04 *	3.74 ± 0.07
TPC-B	44.68	52.19 ± 0.42	57.91 ± 1.15 *
FRAP-B	533.25	647.07 ± 2.95	640.96 ± 26.06
DPPH-B	227.34	246.86 ± 17.99	280.8 ± 14.95
AAC-B	9.07	9.15 ± 0.06 *	7.8 ± 0.14
TPC-C	42.10	43.93 ± 1.09	47.32 ± 1.43 *
FRAP-C	256.51	288.37 ± 3.74	287.44 ± 9.9
DPPH-C	124.29	139.63 ± 3.42	135.32 ± 4.04
AAC-C	6.93	7.92 ± 0.32 *	5.33 ± 0.09

Values represent mean ± standard deviation (*n* = 3). Asterisks (*) indicate statistically significant differences between PLE and STE (*p* < 0.05). TPC in mg GAE/g dw; FRAP in μmol AAE/g dw; DPPH in μmol AAE/g dw; AAC in mg AA/g dw.

**Table 6:** Quantification of identified polyphenolic compounds (mg/g dw) in three Asteraceae species extracted via optimized PLE and conventional STE.

A/A	Polyphenolic Compound	*S. virgaurea*		*T. farfara*		*H. stoechas*	
		PLE Optimal	STE	PLE Optimal	STE	PLE Optimal	STE
1	Gallic acid	0.16 ± 0.01	0.23 ± 0.02 *	n.d.	n.d.	n.d.	n.d.
2	Protocatechuic acid	0.13 ± 0.01	0.26 ± 0.01 *	0.17 ± 0.01	0.29 ± 0.02 *	n.d.	n.d.
3	Neochlorogenic acid	0.54 ± 0.02 *	0.16 ± 0.01	0.24 ± 0.01 *	0.15 ± 0.01	0.41 ± 0.02 *	0.13 ± 0.01
4	Caffeoyl quinic acid derivative 1	n.d.	n.d.	1.23 ± 0.04	1.46 ± 0.05	n.d.	n.d.
5	Chlorogenic acid	1.19 ± 0.05	2.49 ± 0.08 *	0.56 ± 0.03	1.18 ± 0.04 *	1.05 ± 0.04	2.01 ± 0.06 *
6	Caffeoyl quinic acid derivative 2	0.51 ± 0.02	0.46 ± 0.02	0.85 ± 0.03	1.46 ± 0.05 *	0.33 ± 0.01 *	0.02 ± 0.01
7	Caffeoyl quinic acid derivative 3	n.d.	n.d.	0.92 ± 0.03 *	0.43 ± 0.02	0.05 ± 0.01	0.07 ± 0.01
8	*p*-Coumaric acid	n.d.	n.d.	n.d.	n.d.	0.06 ± 0.01	0.11 ± 0.01 *
9	Caffeoyl quinic acid derivative 4	n.d.	n.d.	0.66 ± 0.02	n.d.	n.d.	n.d.
10	Quercetin 3-D-galactoside	0.29 ± 0.01	0.48 ± 0.02 *	n.d.	0.33 ± 0.01	0.11 ± 0.01	0.15 ± 0.01
11	Apigenin derivative	0.27 ± 0.01	n.d.	1.42 ± 0.05	3.31 ± 0.07 *	n.d.	n.d.
12	Caffeoyl quinic acid derivative 5	n.d.	n.d.	1.33 ± 0.04	2.77 ± 0.06 *	0.21 ± 0.01	n.d.
13	Caffeoyl quinic acid derivative 6	n.d.	n.d.	0.56 ± 0.02	1.42 ± 0.04 *	0.60 ± 0.02	2.28 ± 0.05 *
14	Kaempferol-3-glucoside	1.53 ± 0.06	2.53 ± 0.07 *	n.d.	n.d.	1.35 ± 0.05	2.26 ± 0.06 *
15	Apigenin-7-*O*-glucoside	n.d.	n.d.	n.d.	n.d.	0.53 ± 0.02	0.71 ± 0.03 *
16	Caffeoyl quinic acid derivative 7	n.d.	n.d.	0.93 ± 0.03	1.43 ± 0.05 *	0.67 ± 0.03	0.77 ± 0.03 *
17	Apigenin	n.d.	n.d.	n.d.	n.d.	0.32 ± 0.01	0.49 ± 0.02 *
18	Kaempferol	n.d.	n.d.	n.d.	n.d.	0.08 ± 0.01 *	0.01 ± 0.01
	Total identified	4.45 ± 0.12	6.39 ± 0.15 *	9.02 ± 0.21	14.45 ± 0.26 *	5.78 ± 0.18	9.01 ± 0.22 *

Values represent mean ± standard deviation (*n* = 3). Asterisks (*) indicate statistically significant differences between PLE and STE (*p* < 0.05). n.d. means “not detected”.

## Data Availability

The original contributions presented in this study are included in the article, and further inquiries can be directed to the corresponding author.

## Abbreviations

The following abbreviations are used in this manuscript:

AAC	Ascorbic Acid Content
AAE	Ascorbic Acid Equivalents
dw	Dry Weight
FRAP	Ferric-Reducing Antioxidant Power
GAE	Gallic Acid Equivalents
HPLC	High-Performance Liquid Chromatography
MCA	Multivariate Correlation Analysis
PCA	Principal Component Analysis
PLE	Pressurized Liquid Extraction
PLS	Partial Least Squares
RSM	Response Surface Methodology
STE	Stirring Extraction
TCA	Trichloroacetic Acid
TPC	Total Polyphenolic Content
VIP	Variable Importance in Projection

## Appendix A

**Table A1:** Equation of calibration curves for each compound identified through HPLC-DAD.

Polyphenolic Compound	Equation (Linear)	R^2^	RT (min)	UVmax (nm)	LOD (mg/L)	LOQ (mg/L)
Gallic acid	y = 175,167.40x + 105,392.82	0.999	4.723	270	2.87	8.69
Protocatechuic acid	y = 21,282.98x − 63,918.97	0.977	13.400	260	1.74	5.26
Neochlorogenic acid	y = 28,213.52x + 551.72	0.999	16.498	324	2.84	8.61
Chlorogenic acid	y = 50,320.40x − 23,038.36	0.994	21.947	325	2.54	7.71
*p*-Coumaric acid	y = 120,568.59x + 1059.04	0.999	30.082	309	0.97	2.94
Quercetin 3-D-galactoside	y = 41,489.69x − 35,577.55	0.993	34.598	360	3.67	11.11
Kaempferol-3-glucoside	y = 50,916.85x − 42,398.80	0.996	38.724	347	2.31	6.99
Apigenin-7-*O*-glucoside	y = 64,742.65x + 15,897.94	0.998	39.854	336	1.18	3.59
Apigenin	y = 95,483.53x − 5214.26	0.998	55.860	336	0.75	2.29
Kaempferol	y = 93,385.02x − 18,613.03	0.999	56.883	347	0.75	2.29

## References

[1] Fomo G., Madzimbamuto T.N., Ojumu T.V. (2020). Applications of Nonconventional Green Extraction Technologies in Process Industries: Challenges, Limitations and Perspectives. Sustainability.

[2] Rodriguez S., Kaur K., Sharma M. (2025). Extraction Methods and Bioactive Compounds from Pomegranate Peels: A Comprehensive Review for Sustainable Packaging Applications. Food Biomacromol..

[3] Martinez A.S., Lanaridi O., Stagel K., Halbwirth H., Schnürch M., Bica-Schröder K. (2023). Extraction Techniques for Bioactive Compounds of Cannabis. Nat. Prod. Rep..

[4] Anjali K.U., Kamatchi A.R., Haripriya S. (2026). Pressurized Liquid Extraction: Maximizing Extraction Efficiency. Non-Thermal Approaches in Extraction of Bioactive Compounds from Diverse Food Sources.

[5] Athanasiadis V., Mantiniotou M., Kalompatsios D., Makrygiannis I., Alibade A., Lalas S.I. (2024). Evaluation of Antioxidant Properties of Residual Hemp Leaves Following Optimized Pressurized Liquid Extraction. AgriEngineering.

[6] Picot-Allain C., Mahomoodally M.F., Ak G., Zengin G. (2021). Conventional Versus Green Extraction Techniques—A Comparative Perspective. Curr. Opin. Food Sci..

[7] Wu Q., Zhao D., Xiang J., Zhang M., Zhang C., Xu X. (2016). Antitussive, Expectorant, and Anti-Inflammatory Activities of Four Caffeoylquinic Acids Isolated from *Tussilago farfara*. Pharm. Biol..

[8] Rolnik A., Olas B. (2021). The Plants of the Asteraceae Family as Agents in the Protection of Human Health. Int. J. Mol. Sci..

[9] Horozić E., Kolarević L., Huseinović E., Husejnagic D., Merima I., Karic E., Cilović Kozarević E., Cipurkovic S. (2023). *Tussilago farfara* L.: Antioxidant and Antibacterial Potential of Extracts in In Vitro Conditions. Int. J. Basic Appl. Sci..

[10] Bota V.B., Neamtu A.-A., Olah N.-K., Chișe E., Burtescu R.F., Furtuna F.R.P., Nicula A.-S., Neamtu C., Maghiar A.-M., Ivănescu L.-C. (2022). A Comparative Analysis of the Anatomy, Phenolic Profile, and Antioxidant Capacity of *Tussilago farfara* L. Vegetative Organs. Plants.

[11] Sharma M., Navneet B., Sharma M. (2023). Ethnobotany, Phytochemistry, Pharmacology and Nutritional Potential of Medicinal Plants from Asteraceae Family. J. Mt. Res..

[12] Bakar F., Bahadır Acıkara Ö., Ergene B., Nebioğlu S., Saltan Çitoğlu G. (2015). Antioxidant Activity and Phytochemical Screening of Some Asteraceae Plants. Turk. J. Pharm. Sci..

[13] Majeed S., Bibi F., Khan Y., Rubab S., Zafar M. (2025). Ethnobotanical Overview of Selected Asteraceae Species. Trans. Inst. Mol. Biol. Biotechnol..

[14] Barroso M.R., Barros L., Dueñas M., Carvalho A.M., Santos-Buelga C., Fernandes I.P., Barreiro M.F., Ferreira I.C.F.R. (2014). Exploring the Antioxidant Potential of *Helichrysum stoechas* (L.) Moench Phenolic Compounds for Cosmetic Applications: Chemical Characterization, Microencapsulation and Incorporation into a Moisturizer. Ind. Crops Prod..

[15] Kherbache A., Senator A., Laouicha S., Al-Zoubi R.M., Bouriche H. (2020). Phytochemical Analysis, Antioxidant and Anti-Inflammatory Activities of *Helichrysum stoechas* (L.) Moench Extracts. Biocatal. Agric. Biotechnol..

[16] Fursenco C., Calalb T., Uncu L., Dinu M., Ancuceanu R. (2020). *Solidago virgaurea* L.: A Review of Its Ethnomedicinal Uses, Phytochemistry, and Pharmacological Activities. Biomolecules.

[17] Kraujalienė V., Pukalskas A., Venskutonis R. (2017). Biorefining of Goldenrod (*Solidago virgaurea* L.) Leaves by Supercritical Fluid and Pressurized Liquid Extraction and Evaluation of Antioxidant Properties and Main Phytochemicals in the Fractions and Plant Material. J. Funct. Foods.

[18] Vojvodić S., Božović D., Aćimović M., Gašić U., Zeković Z., Bebek Markovinović A., Bursać Kovačević D., Zlatković B., Pavlić B. (2025). A Preliminary Insight into Under-Researched Plants from the Asteraceae Family in the Balkan Peninsula: Bioactive Compound Diversity and Antioxidant Potential. Plants.

[19] Mustafa A., Turner C. (2011). Pressurized Liquid Extraction as a Green Approach in Food and Herbal Plants Extraction: A Review. Anal. Chim. Acta.

[20] Cano-Lamadrid M., Martínez-Zamora L., Mozafari L., Bueso M.C., Kessler M., Artés-Hernández F. (2023). Response Surface Methodology to Optimize the Extraction of Carotenoids from Horticultural By-Products—A Systematic Review. Foods.

[21] Liu Y., Dar B.N., Makroo H.A., Aslam R., Martí-Quijal F.J., Castagnini J.M., Amigo J.M., Barba F.J. (2024). Optimizing Recovery of High-Added-Value Compounds from Complex Food Matrices Using Multivariate Methods. Antioxidants.

[22] Lee L.C., Liong C.-Y., Jemain A.A. (2018). Partial Least Squares-Discriminant Analysis (PLS-DA) for Classification of High-Dimensional (HD) Data: A Review of Contemporary Practice Strategies and Knowledge Gaps. Analyst.

[23] Box G.E.P., Draper N.R. (2007). Response Surfaces, Mixtures, and Ridge Analyses.

[24] Box G.E.P., Behnken D.W. (1960). Some New Three Level Designs for the Study of Quantitative Variables. Technometrics.

[25] Mantiniotou M., Athanasiadis V., Kalompatsios D., Bozinou E., Ntourtoglou G., Dourtoglou V.G., Lalas S.I. (2025). Atmospheric Room Temperature Plasma as a Green Pretreatment Strategy for Enhanced Phytochemical Extraction from *Moringa oleifera* Leaves. Foods.

[26] Kalompatsios D., Athanasiadis V., Mantiniotou M., Lalas S.I. (2024). Optimization of Ultrasonication Probe-Assisted Extraction Parameters for Bioactive Compounds from *Opuntia macrorhiza* Using Taguchi Design and Assessment of Antioxidant Properties. Appl. Sci..

[27] Chaabani E., Abert Vian M., Bettaieb Rebey I., Bourgou S., Zar Kalai F., Chemat F., Ksouri R. (2023). Ethanol–Water Binary Solvent Affects Phenolic Composition and Antioxidant Ability of *Pistacia lentiscus* L. Fruit Extracts: A Theoretical Versus Experimental Solubility Study. J. Food Meas. Charact..

[28] Catena S., Rakotomanomana N., Zunin P., Boggia R., Turrini F., Chemat F. (2020). Solubility Study and Intensification of Extraction of Phenolic and Anthocyanin Compounds from *Oryza sativa* L. ‘Violet Nori’. Ultrason. Sonochem..

[29] Kalra R., Conlan X.A., Goel M. (2020). Fungi as a Potential Source of Pigments: Harnessing Filamentous Fungi. Front. Chem..

[30] Plaskova A., Mlcek J. (2023). New Insights of the Application of Water or Ethanol-Water Plant Extract Rich in Active Compounds in Food. Front. Nutr..

[31] Višnjevec A.M., Barp L., Lucci P., Moret S. (2024). Pressurized Liquid Extraction for the Determination of Bioactive Compounds in Plants with Emphasis on Phenolics. TrAC Trends Anal. Chem..

[32] Mantiniotou M., Athanasiadis V., Liakos K.G., Bozinou E., Lalas S.I. (2025). Artificial Intelligence and Extraction of Bioactive Compounds: The Case of Rosemary and Pressurized Liquid Extraction. Processes.

[33] Yavorska H.V., Vorobets N.M. (2025). Evaluation of Antifungal Activity of “Green” *Solidago canadensis* Extracts. J. VNKarazin Kharkiv Natl. Univ. Ser. Biol..

[34] Abdel-Gawad A.I.M., Farid M.H., Soliman M.N.A., Sheta M.H., Zarad M.M., Bakr A.M., Nada R.S., Hassan A.S., Atta R.F., Ghoneim A.H. (2025). A Comparative Study of Conventional and Nano-NPK on the Growth, Flowering, Bioactive Compounds, and Anatomical Characters of *Solidago virgaurea*. Egypt. J. Soil Sci..

[35] Marques M.P., Landim E., Varela C., da Costa R.M.F., Marques J., Batista de Carvalho L.A.E., Silva A., Cruz M.T., André R., Rijo P. (2025). A Spectrochemically Driven Study: Identifying Phenolic-Rich Extracts from *Helichrysum stoechas*, *Lavandula pedunculata*, and *Thymus mastichina* with Potential to Revert Skin Aging Effects. Pharmaceuticals.

[36] Hwang S.H., Kim J.H., Wang Z., Lee J.-Y., Lim S.S. (2017). Analytical Method for the Validation of Three Polyphenols as a Marker Compound for the Standardization of *Solidago virgaurea* Subsp. *gigantea* Extracts and Antiadipogenesis of Harvesting Time and Location. J. Anal. Methods Chem..

[37] Athanasiadis V., Avdoulach-Chatzi-Giousouf E., Koulouri E., Kalompatsios D., Lalas S.I. (2025). Data-Driven Optimization of Polyphenol Recovery and Antioxidant Capacity from Medicinal Herbs Using Chemometrics and HPLC Profiling for Functional Food Applications. Int. J. Mol. Sci..

